# Editorial: Recent advances in image fusion and quality improvement for cyber-physical systems, volume II

**DOI:** 10.3389/fnbot.2024.1422982

**Published:** 2024-05-29

**Authors:** Xin Jin, Shin-Jye Lee, Michal Wozniak, Qian Jiang

**Affiliations:** ^1^School of Software, Yunnan University, Kunming, Yunnan, China; ^2^Department of Systems and Computer Networks, Faculty of Information and Communication Technology, Wroclaw University of Science and Technology, Wroclaw, Poland; ^3^Institute of Management of Technology, National Yang Ming Chiao Tung University, Hsinchu, Taiwan

**Keywords:** artificial neural networks, embedded learning system, feature extraction, image quality improvement, image fusion, robot vision

Multi-source visual information fusion and quality improvement can help the robotic system to perceive the real world. Image fusion is a computational technique fusing multi-source images from multiple sensors into a synthesized image that provides a comprehensive or reliable description. Quality improvement techniques can be used to address the challenge of low-quality image analysis tasks (Jin et al., 2017, 2023a, 2024; Liu et al., 2022; Wang G. et al., 2022; Guo et al., 2024). At present, a lot of brain-inspired algorithm methods (or models) are aggressively proposed to accomplish these two tasks, and the artificial neural network has become one of the most popular techniques in processing image fusion and quality improvement techniques in this decade, especially deep convolutional neural networks (Kong et al., 2022; Liu et al., 2022; Wang G. et al., 2022; Chen et al., 2023; Jin et al., 2023a). This is an exciting research field for the research community of image fusion, and many interesting Research Topics remain to be explored, such as deep few-shot learning, unsupervised learning, application of embodied neural systems, and industrial applications.

How to develop a sound biological neural network and embedded system to extract the multiple features of source images are two key questions that need to be addressed in the fields of image fusion and quality improvement. Hence, studies in this field can be divided into two aspects: new end-to-end neural network models for merging constituent parts during the image fusion process and the embodiment of artificial neural networks for image processing systems. In addition, current booming techniques, including deep neural systems and embodied artificial intelligence systems, are considered potential future trends for reinforcing image fusion performance and quality improvement (Wang W. et al., 2022; Yang et al., 2022; Zhang et al., 2022, 2023; Jin et al., 2023b; Liu et al., 2023; Mi et al., 2023).

The paper of Zhang et al. introduces a palmprint recognition method based on a gating mechanism and adaptive feature fusion. They propose a new network structure, GLGAnet, for extracting local and global features of palmprints. The method incorporates a gating mechanism to control features extracted by deep convolutional layers and Transformer modules, along with an adaptive convolution fusion module for multi-level feature fusion. Experimental results demonstrate that their method outperforms existing approaches on two datasets.

Many previous works overlooked the crucial support-query set interaction and the deeper information that needs to be explored. Zeng et al. propose a duplex network model utilizing the suppression and focus concept to address this issue. Their network includes dynamic convolution, prototype matching structure, and a hybrid attention module called DAAConv. The DPMCN model demonstrates superior performance over traditional prototype-based methods in dataset experiments.

In the third work, Peng et al. proposed a network structure called Context-Aware Lightweight Super-Resolution Network, which enhances the resolution of remote sensing images. This network combines local and global features and includes a Dynamic Weight Generation Branch to improve image quality while maintaining computational efficiency. Compared to existing methods, the proposed approach can reconstruct high-quality images at a lower cost.

In the fourth study, titled “*Feature fusion network based on few-shot fine-grained classification*,” Yang et al. introduced the Feature Fusion Similarity Network (FFSNet). This model employs global measures to accentuate the differences between classes while utilizing local measures to consolidate intra-class data, greatly enhancing the model's generalization ability. The method proposed in this paper has been validated to be effective.

In the fifth work, Chen et al. introduced ID-YOLOv7, a convolutional neural network specifically designed for insulator defect detection in power distribution networks, by introducing several novel methods, including Edge Detailed Shape Data Augmentation, Cross-Channel and Spatial Multi-Scale Attention module and Re-BiC module, to enhance the model's sensitivity to subtle defect features and fuse multi-scale contextual features. Experimental results demonstrate that the algorithm significantly boosts detection performance.

## Author contributions

XJ: Writing – original draft. S-JL: Writing – review & editing. MW: Writing – review & editing. QJ: Writing – review & editing, Conceptualization.
